# A study protocol for a Pilot Masked, Randomized Controlled Trial Evaluating Locally-applied Gentamicin versus Saline in Open Tibia Fractures (pGO-Tibia) in Dar es Salaam, Tanzania

**DOI:** 10.1186/s40814-021-00766-7

**Published:** 2021-02-10

**Authors:** Ericka P. von Kaeppler, Claire Donnelley, Syed H. Ali, Heather J. Roberts, John M. Ibrahim, Hao-Hua Wu, Edmund N. Eliezer, Travis C. Porco, Billy T. Haonga, Saam Morshed, David W. Shearer

**Affiliations:** 1 Department of Orthopaedic Surgery, Institute for Global Orthopaedics and Traumatology, University of California San Francisco School of Medicine, 2550 23rd Street, Building 9, 2nd Floor, San Francisco, CA 94110 USA; 2 Muhimbili Orthopaedic Institute, Muhimbili National Hospital, Kalenga Street, Dar es Salaam, Tanzania; 3 F.I. Proctor Foundation, University of California San Francisco, 513 Parnassus Avenue, San Francisco, CA 94122 USA; 4 Department of Ophthalmology, University of California San Francisco, 10 Koret Way, San Francisco, CA 94143 USA; 5 Department of Epidemiology and Biostatistics, University of California San Francisco, 550 16th St. 2nd Floor, San Francisco, CA 94158 USA

**Keywords:** Global health, Orthopaedic surgery, Fracture-related infection, Open tibia fracture, Gentamicin, Antibiotics

## Abstract

**Background:**

Open tibia fractures are a major source of disability in low- and middle-income countries (LMICs) due to the high incidence of complications, particularly infection and chronic osteomyelitis. One proposed adjunctive measure to reduce infection is prophylactic local antibiotic delivery, which can achieve much higher concentrations at the surgical site than can safely be achieved with systemic administration. Animal studies and retrospective clinical studies support the use of gentamicin for this purpose, but no high-quality clinical trials have been conducted to date in high- or low-income settings.

**Methods:**

We describe a protocol for a pilot study conducted in Dar es Salaam, Tanzania, to assess the feasibility of a single-center masked randomized controlled trial to compare the efficacy of locally applied gentamicin to placebo for the prevention of fracture-related infection in open tibial shaft fractures.

**Discussion:**

The results of this study will inform the design and feasibility of a definitive trial to address the use of local gentamicin in open tibial fractures. If proven effective, local gentamicin would be a low-cost strategy to reduce complications and disability from open tibial fractures that could impact care in both high- and low-income countries.

**Trial registration:**

ClinicalTrials.gov, Registration # NCT03559400; Registered June 18, 2018.

**Supplementary Information:**

The online version contains supplementary material available at 10.1186/s40814-021-00766-7.

## Background

Musculoskeletal trauma is a source of significant morbidity and mortality in low- and middle-income countries (LMICs) [1]. Compared to high-income countries (HIC), LMICs experience both a greater burden of trauma [1] and higher infection risk following surgical treatment of traumatic injuries [2–4]. Within musculoskeletal injury in LMICs, open tibia fractures represent a disproportionate morbidity burden due to the high risk of infection and other postoperative complications [5]. While prompt parenteral antibiotic administration, thorough surgical debridement, and fracture stabilization are known to mitigate risk, open tibia fractures remain highly susceptible to postoperative infection. One proposed adjunctive measure to reduce fracture-related infections (FRIs) is prophylactic local antibiotic delivery. Intrawound antibiotics can achieve much higher concentrations at the surgical site than can safely be achieved with systemic administration [6, 7] due to impaired blood supply at the site of injury and risk of systemic toxicity [8]. Despite demonstrated reductions in deep surgical site infections of up to 70% with local antibiotic administration [9], the growing body of literature evaluating local antibiotics at the time of wound closure is heterogeneous, is primarily retrospective, and relies on poorly defined infection classification criteria (Kim et al., submitted for publication). Further, much of the existing literature neither originates from nor addresses LMIC-specific needs such as being low cost, readily available, and easy to administer. As such, high-quality, LMIC-targeted studies are needed to determine the clinical effectiveness and appropriateness of local antibiotic administration in addressing the high burden of open tibia fracture-associated infection in LMICs.

### Aims and objectives

We present a study protocol for a masked, randomized, placebo-controlled trial to compare the efficacy of intraoperative, locally applied gentamicin to placebo saline injection for the prevention of fracture-related infection. The aim of this single-center study is to evaluate feasibility of an adequately powered definitive trial to address the burden of infection after open tibia fractures. Feasibility outcomes include rate of enrollment, retention, and data completeness of key outcomes. Additionally, evaluation of planned primary and secondary endpoints which are intended to be used in a later, definitive trial will be performed in order to refine estimates of effect size. The planned primary endpoint of the definitive trial is the occurrence of fracture-related infection at 1 year. Planned secondary endpoints of the definitive trial are (1) occurrence of nonunion, (2) occurrence of unplanned fracture-related reoperation, (3) health-related quality of life, (4) radiographic healing, (5) clinical healing, (6) occurrence of suggestive fracture-related infection criteria, and (7) economic impact as measured by direct medical costs and lost productivity associated with injury. We hypothesize that the risk of fracture-related infection will be reduced by the intraoperative use of locally administered gentamicin at the time of wound closure.

### Study site and collaboration

The efficacy of local antibiotic administration in the prevention of fracture-related infection in open tibial shaft fractures is best addressed in a high-volume setting. Muhimbili Orthopaedic Institute (MOI) is the largest referral hospital for orthopaedic trauma care in Tanzania and among the busiest in East Africa, admitting 459 injured patients per month.

The coordinating center and MOI have successfully collaborated on previous randomized clinical trials, including a trial of open tibia fractures that enrolled and randomized 240 patients in 16 months, a higher number of patients than has been achieved by any single center in multiple clinical trials conducted in North America and India (Table 1). The development and dissemination of this new protocol therefore builds upon shared experience to outline the methods and standard operating procedures for a masked randomized controlled trial in a resource-limited setting. This includes developing masking and randomization protocols, establishing fracture- and LMIC-relevant outcome measures, standardizing local antibiotic injection [6], and testing the potency of antibiotics obtained in an LMIC setting.

**Table 1:** Published clinical trials reporting outcomes of open tibial shaft fractures

Studies	Total sample	Open tibia sample	Number centers	Duration (years)	Enrolled/center	Enrolled/center/year	Follow-up rate
FLOW Trial [10]	2447	912	41	4	22	6	90%
SPRINT Trial [11]	1226	392	29	5	13	3	93%
**MOI IMN v. Ex-fix** **[**12]	**240**	**240**	**1**	**1.3**	**240**	**180**	**92.1%**
TRUST Trial [13]	501	114	43	4.5	3	< 1	96%
LEAP Study [14]	569	173	8	3	22	7	96%
INFINITI [15]	768	162	9	NR	18	NR	98%
Alberta Cohort [16]	791	140	3	8	47	6	94%

Bold face text represents the previous randomized trial conducted by the coordinating center and MOI that enrolled and randomized 240 patients in 16 months

### Gentamicin rationale

An ideal local antibiotic for open fractures is (1) low cost, (2) readily available, (3) easily applied, (4) easily monitored for toxicity, and (5) active against the microbiological profile of the wound. The most common pathogens in fracture-related infection are *Staphylococcus aureus*, followed by *Staphylococcus epidermidis*, and gram-negative bacteria such as *Escherichia coli* and other Enterobacteriaceae species [17, 18]. Gentamicin provides excellent coverage of these species with low rates of resistance, particularly when combined with systemic administration of a first-generation cephalosporin [19]. In HICs, antibiotic bead or intrawound vancomycin use is common, but their use in LMICs is limited by relatively a high cost, a narrow antimicrobial spectrum, and the potential for inducing drug resistance [20]. Aqueous local gentamicin is a promising alternative for LMIC settings.

Gentamicin covers common pathogens implicated in FRI [21], is widely available and inexpensive [9], and has low systemic toxicity compared to systemic aminoglycoside use which carries concern of acute kidney injury due to combined effects of traumatic muscle breakdown and aminoglycoside toxicity. Systemic toxicity was not reported in any gentamicin study included in a recent systematic review of local antibiotics in spine and trauma surgery [9] (Kim et al., submitted for publication). If renal toxicity does occur with locally administered gentamicin, it can be easily and inexpensively monitored with serum creatinine, a routine laboratory measurement [22]. In addition, Miclau et al. and others have shown that aminoglycosides have no significant effect on fracture healing both in vitro and in small animal models [23, 24]. One prior retrospective study evaluating aminoglycosides in open tibia fractures reported a 2.7-fold decrease in the unadjusted odds of deep infection without adverse effects [6]. Finally, gentamicin enables intraoperative surgeon masking in this placebo-controlled trial as it appears clear in liquid form and cannot be visually distinguished from normal saline.

## Methods

### Study design

The Pilot Masked, Randomized Controlled Trial Evaluating Locally-applied Gentamicin versus Saline in Open Tibia Fractures (pGO-Tibia) study is a single-center, masked, individually randomized, placebo-controlled trial to evaluate the feasibility of a definitive clinical trial investigating the efficacy of intraoperative local gentamicin for prevention of fracture-related infection in open tibial shaft fractures.

The protocol was developed in accordance with recommendations from the Standard Protocol Items: Recommendations for Interventional Trials (SPIRIT) [25] guidelines as well as the Consolidated Standards of Reporting Trials (CONSORT) guidelines for randomized pilot and feasibility trials [26]. Using the PRagmatic Explanatory Continuum Indicator Summary (PRECIS-2) tool [27] (Table 2), the trial was determined to contain both pragmatic and explanatory elements, but ultimately tended towards a more pragmatic intention.

**Table 2:** PRECIS score

Domain	Score	Rationale
Eligibility	3	Eligibility criteria include all open tibial shaft fractures, but exclude people (a) with delayed presentation, (b) unlikely to follow-up, and (c) with significant associated injuries (TBI, burns, bilateral fractures).
Recruitment	5	No recruitment outside of ‘usual care’ occurs.
Setting	4	Study is conducted at a center in which the results are intended to be applied but is conducted a single center only.
Organization	3	Study intervention is done in the course of ‘usual care’ without need for increased care providers, but the use of intraoperative local antibiotic represents an increased cost and service above ‘usual care’.
Flexibility (delivery)	3	Though minimal training is needed for administering surgeons, correct injection protocol must be followed in order to achieve ideal placement of active agent.
Flexibility (adherence)		N/A according to PRECIS-2 guidelines because after intraoperative intervention, no further adherence is required.
Follow-up	3	Study follow-up includes additional follow-up visits and collection of additional data above ‘usual care’.
Primary outcome*	5	The primary outcome is of great interest to both patients and health care providers and does not require expertise beyond the treating physician for diagnosis.
Primary analysis	5	All available data will be used according to the intention-to-treat principle.

### Study setting

The study setting for the pGO-Tibia randomized controlled pilot trial is MOI in Dar es Salaam, Tanzania. MOI is a tertiary referral hospital with a large catchment area and high volume of adult musculoskeletal trauma that, combined with its strong leadership and prior trial experience, has the capacity to manage this large-scale RCT. A trial steering committee comprised of local principal investigators (BH, EE) and coordinating center principal investigators (DS, SM) oversees the protocol development and implementation. Patient and participant involvement was not sought out for the purposes of this study.

The pGO-Tibia trial is registered at ClinicalTrials.gov (NCT03559400) on June 18, 2018 (https://clinicaltrials.gov/ct2/show/NCT03559400). The trial is funded by the following sources:

- Orthopaedic Trauma Association International Grant (Grant ID# 4279)
- Hellman Fellows Fund awarded by the UCSF Hellman Fellowship Program (Grant ID# A7029503)
- SuperNOVA awarded by UCSF Department of Orthopaedic Surgery

### Ethical approval

Ethical approval for this trial has been obtained from the National Institute of Medical Research, Tanzania (Ref#: NIMR/HQ/R.8a/Vol. IX/2958) and the coordinating center’s Human Subjects Research Internal Review Board (IRB# 17-23950, Ref#: 260102). Any proposed protocol modifications will be submitted for approval to both ethical review boards.

### Recruitment strategy and participant characteristics

All patients presenting to MOI ≥ 18 years old with an open tibia fracture are screened for eligibility (Table 3). Eligible patients are approached to participate in the trial and may be enrolled following written, informed consent as obtained by trained research coordinators. All patients who undergo screening are administered systemic ceftriaxone for open fracture prophylaxis [28] regardless of whether they are ultimately found eligible or choose to participate. In addition, preoperative serum creatinine levels and radiographs are recorded per standard institutional protocol. Recruitment for this pilot study began in November 2019 and is expected to continue through December 2020.

**Table 3:** Inclusion and exclusion criteria

Inclusion criteria	Exclusion criteria
1. Age ≥ 18 years	1. Time from injury to presentation > 48 h
2. Open tibial shaft fracture meeting the following criteria:	2. Time from injury to surgery > 7 days
a. OTA type 42	3. Aminoglycoside allergy
b. Primarily closable wound	4. GA IIIB or IIIC open fractures
c. GA I, II, or IIIA	5. Bilateral open tibia fractures
	6. Severe brain (GCS < 12) or spinal cord injury
	7. Severe vascular injury
	8. Severe burns (> 10% TBSA or > 5% TBSA with full thickness or circumferential injury)
	9. Pathologic fracture
	10. History of ipsilateral active limb infection
	11. Unlikely to complete follow-up

*Abbreviations*: *OTA* Orthopaedic Trauma Association, *GA* Gustilo-Anderson, *GCS* Glasgow Coma Scale, *TBSA* total body surface area

### Treatment group allocation

All consenting patients are treated per institutional protocol with urgent debridement and bony stabilization with either internal or external fixation based on surgeon preference. Patients with wounds determined intraoperatively to not be amenable to primary closure are excluded. If the wound is closed primarily, participants are randomized by research coordinators to receive intraoperative local injection of either aqueous gentamicin solution (intervention) or normal saline solution (placebo control) administered immediately following wound closure. If at the conclusion of the procedure, the treating surgeon is unable to close the wound primarily, the patient is excluded from the study prior to randomization.

Allocation to the study groups is performed using a web-based randomization tool as part of Research Electronic Data Capture (REDCap) [29, 30]. The randomization sequence was generated using randomly permutated blocks of 4, 6, and 8 with a 1:1 allocation ratio.

### Masking

Masking is established and maintained by preparation of the study solutions in visually indistinguishable syringes labeled either “Solution A” or “Solution B”. One coordinating center research personnel is unmasked for the implementation and management of trial protocols, and the certified study nurse responsible for preparation of study solutions at MOI is unmasked. These unmasked individuals neither has any contact with study participants nor is aware of the treatment group to which each participant is assigned.

Trial participants are masked to the treatment group allocation. All health care providers involved in care of the trial participants including physicians, surgeons, and nurses are masked to treatment group allocation. Research team members including research coordinators, data collectors, and data analysts are masked to treatment group allocation.

### Study solution preparation, storage, and quality control

**Fig. 1:**
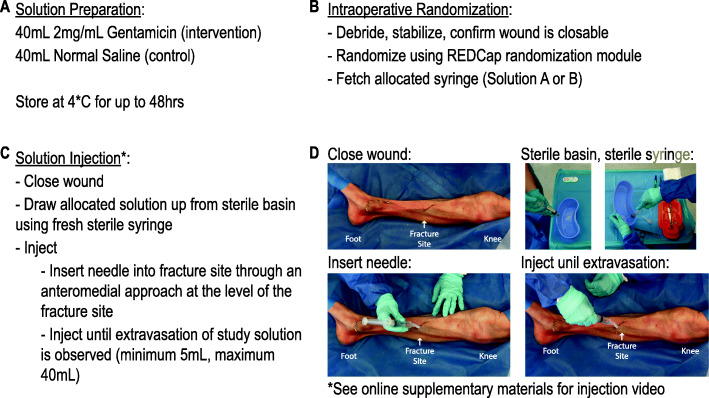
Study Intervention Protocol. **a** The preparation and storage of 40 mL study solutions, either 2 mg/mL gentamicin (intervention) or normal saline (placebo control). **b** The process of intraoperative randomization. Following fracture debridement and bony stabilization with planned fixation, the surgeon confirms that the wound amenable to primary closure. If the wound is primarily closable, participants are randomized to receive intraoperative local injection of either intervention or placebo control, labeled either ‘Solution A’ or ‘Solution B’. Research coordinators retrieve the allocated syringe of study solution from the dedicated study refrigerator. **c** The process of solution administration. Following wound closure, the surgeon draws the study solution from a sterile basin into a sterile syringe and injects the study solution by inserting the needle down to bone through an anteromedial approach at the level of the fracture site. A minimum of 5 mL of study solution may be injected, and the injection is continued until either extravasation is seen through the traumatic wound or a maximum of 40 mL has been administered, whichever occurs first. **d** The process of solution administration. A full-video demonstration of injection procedure can be found in Additional file 1

Aqueous gentamicin and normal saline solutions are prepared by the certified study nurse using sterile technique. The gentamicin solution consists of 2 mg/mL aqueous gentamicin (Sichuan Long March Pharmaceutical Co., Ltd., Leshan, Sichuan Province, China) while the control solution consists of normal saline (Otsuka Pharmaceutical India Private Limited, Ahmedabad, India) without active agent. The gentamicin dose was chosen based on a previously published study [6]. The working solutions are prepared in identical syringes labeled either ‘Solution A’ or ‘Solution B’, according to the masking key, and are labeled with the date of preparation and date of expiration. The study nurse maintains a preparation log to ensure the integrity of the study solutions (Fig. 1a).

Study solutions are stored in a locked, dedicated study refrigerator at 4 °C for up to 48 h, in accordance with pharmacist guidelines. The study refrigerator is checked daily by the study nurse, and a dedicated use log is maintained to ensure the integrity of the study solution storage and usage.

For quality control, the efficacy of masked study solutions is tested against standard organisms by the Muhimbili University of Health and Allied Sciences (MUHAS) microbiology lab, with results evaluated by unmasked study personnel. This occurs once per month during the first 6 months and once every 6 months thereafter.

### Surgical technique and intervention administration

The study protocol does not influence the surgical plan for debridement or stabilization of the open fracture. Determination of method of fracture debridement and stabilization is left to the discretion and best practices of the operating surgeon and may consist of either intramedullary nailing or external fixation. Type of fixation does not affect participant inclusion or study protocol.

Following intraoperative randomization (Fig. 1b), the appropriate study solution is provided to the administering surgeon in a masked manner. The local solution injection is administered immediately following wound closure. As described previously by Lawing et al. [6], the solution is injected by inserting a 22 gauge needle down to bone through an anteromedial approach at the level of the fracture site such that the injected solution fills the wound cavity (Additional file 1). A minimum of 5 mL of study solution may be injected, and the injection is continued until either extravasation is seen through the traumatic wound or a maximum of 40 mL has been administered, whichever occurs first (Fig. 1c, d).

### Potential adverse events associated with gentamicin

One of the main concerns associated with aminoglycoside use is nephrotoxicity, though local administration may reduce the risk compared with systemic administration [8]. Studies have reported little to no toxicity from locally administered antibiotic-impregnated cement beads [31]. Despite the likely low risk of nephrotoxicity from locally administered gentamicin, serum creatinine is measured in every patient preoperatively and on postoperative day 2. If serum creatinine levels on postoperative day 2 meet criteria for acute kidney injury, defined as ≥ 1.5 fold increase or ≥ 26.5 umol/L increase from preoperative baseline [32], serum creatinine levels are redrawn at the 2-week follow-up visit.

To evaluate the concern that local antibiotics administered intraoperatively in the wound at higher concentrations may inhibit bone and tissue healing [33, 34], fracture healing is monitored clinically and radiographically at 6 weeks, 3 months, 6 months, 9 months, and 12 months after surgery for signs of nonunion or malunion associated with local gentamicin administration.

## Outcome measures

### Feasibility outcomes

The feasibility outcomes are [35, 36] (Table 4):

1. Enrollment rate [37–39]: the number of patients enrolled per month will be reported.
2. Retention: the percent of randomized patients who complete 1 year follow-up will be reported. We aim for a follow-up rate greater than 90% at 1 year, which was achieved in our previous open tibia fracture trial performed through the same collaborative partnership [12].
3. Data completeness [37]: the proportion complete at each timepoint of the following indices will be reported:
  - Safety outcomes, including serum creatinine measurement and adverse event screening
  - Treatment group allocation
  - Fracture-related infection (FRI) criteria, a set of consensus diagnostic criteria that includes clinical signs and diagnostic studies for diagnosing fracture-related infection [40]).

**Table 4:** Outcomes analysis

Outcome	Data	Method of analysis
*Feasibility*		
Enrollment rate	# enrolled per month	Descriptive statistics using means, proportions, and variances (no inferential statistics)
Retention	% completing 1-year follow-up	
Data completeness	% complete data, including: • Safety outcomes • Treatment group allocation • FRI criteria	
*Planned primary endpoint*		
Occurrence of fracture-related infection	FRI criteria (time-to-event)	Relative hazard as estimated by two-sided binomial regression with the complementary log-log link, with a type I error rate (alpha) of 0.05.
*Planned secondary endpoints*		
Occurrence of nonunion	Nonunion criteria (binary)	Fisher’s exact test on the 2 × 2 cross-tabulation for binary variables Comparison means of active agent group and placebo group using two-tailed Student’s *t* test (alpha = 0.05) for continuous variables
Occurrence of unplanned reoperation	Review of complications (binary)	
Health-related quality of life	EQ-5D (continuous)	
Radiographic healing	mRUST (continuous)	
Clinical healing	FIX-IT (continuous)	
Suggestive FRI	FRI criteria (binary)	
Lost productivity	WPAI (continuous)	
Direct medical costs	Micro-costing (continuous)	

### Planned primary endpoint

The planned primary endpoint is occurrence of *fracture-related infection* (FRI) during the year of follow-up [40] (Table 4), a binary variable. FRI was selected as the criteria for defining infection as centers for disease control definition of surgical site infection cannot easily be extrapolated to the fracture setting [40]. FRI diagnosis is likely to peak between 3 and 6 months after surgery and has a non-normal time-to-event distribution, with incident cases rarely presenting later than 12 months after surgery. Any of the four following diagnostic criteria are confirmatory for infection: (1) fistula, sinus, or wound breakdown; (2) purulent drainage from the wound or presence of pus during surgery; (3) phenotypically indistinguishable pathogens identified by culture from at least two separate deep tissue/implant specimens; or (4) presence of microorganisms in deep tissue taken during an operative intervention, as seen on histopathological examination.

### Planned secondary endpoints

The planned secondary endpoints are (Table 4):

1. Occurrence of nonunion, a binary variable, as defined by:
  - Any unplanned reoperation for promotion of bone healing; OR
  - Modified Radiographic Union Score for Tibia (mRUST*) [41] score ≤ 10 AND recommendation by treating surgeon for nonunion repair surgery. The mRUST is an ordinal scale of radiographic healing ranging from 4 to 16 [41]. The Function Index for Trauma (FIX-IT) is an ordinal scale of clinical healing from 0 to 12 that encompasses two domains, ability to weight-bear and pain at the fracture site, each scored from 0 to 6 [42]).
2. Occurrence of unplanned fracture-related reoperation, a binary variable, for infection, wound healing, or fracture union, excluding removal of implants for prominence or irritation. This may include but is not limited to:
  - Irrigation and debridement of surgical incisions or open fracture wounds due to infection or wound healing problems
  - Revision wound closure for dehiscence
  - Soft tissue coverage procedure for infected or necrotic wound
  - Surgery for delayed union or nonunion, such as bone grafting or implant exchange
  - Reoperation for implant failure due to infection or bone-healing problems
  - Amputation for infection, wound, or fracture healing problem
3. Radiographic healing via the mRUST score, an ordinal scale ranging from 4 to 16 [41]
4. Clinical healing via the FIX-IT score [42]
5. Health-related quality of life via EuroQol-5 Dimensions, 3-level questionnaire (EQ-5D), a validated health-related quality of life measurement questionnaire [43]
6. FRI suggestive criteria, including clinical (wound redness, fever) and radiographic signs (sequestrum), elevated serum inflammatory markers, and new onset or increased wound drainage [40]
7. Direct medical costs, as measured by micro-costing with direct observation using time and motion analysis and patient chart review [44]. Micro-costing using time and motion analysis is a method of assessing direct medical costs associated with hospitalization [44]. As time and motion analysis is resource-intensive, micro-costing will be performed on a convenience sample of 25% of enrolled patients. Variability in direct costs will be reported and used to inform the percentage of patients in the definitive trial for whom micro-costing should be performed to achieve a reliable estimate of direct costs.
8. Indirect costs, including lost productivity as measured by the Work Productivity and Impairment Assessment: Lower Limb Fracture (WPAI:LLF) [45] and transportation costs for medical care.
9. WPAI:LLF assesses the impact of fracture on four domains: absenteeism (missed work), presenteeism (decreased productivity at work), work productivity loss (accounting for both absenteeism and presenteeism), and activity impairment outside of work [45].

### Subgroup analyses

The following subgroups are identified for future subgroup analyses:

1. Gustilo-Anderson (GA) classification of fracture (type I or II vs. III)
2. Contamination (minimal or moderate vs. severe based on the Orthopaedic Trauma Association Open Fracture Classification)
3. Time to surgery (≤ 24 vs. > 24 h after injury)
4. Type of fixation (external fixation vs. intramedullary nailing).

## Data collection and follow-up

Following participant enrollment and informed consent, baseline clinical and demographic data are collected, including socioeconomic status and baseline WPAI, medical and social history, injury characteristics, and estimated pre-injury health-related quality of life. Contact information for the patient and at least two close contacts is collected to optimize follow-up. Pre- and postoperative radiographs are obtained prior to hospital discharge, and serum creatinine is obtained preoperatively and on postoperative day 2. If serum creatinine levels on postoperative day 2 meet the criteria for acute kidney injury, defined as ≥ 1.5 fold increase or ≥ 26.5 umol/L increase from preoperative baseline [32], serum creatinine levels are redrawn at 2-week follow-up visit. For a subset of patients, direct medical costs during hospitalization are assessed. For the intraoperative setting, resource utilization, personnel involvement, and time data are directly observed and recorded for use in time and motion analysis. Resource utilization throughout the hospital stay is determined from chart review. The schedule of patient encounters and corresponding data to be collected is described in Table 5.

**Table 5:** Schedule of events

	Hospital				Outpatient					
	Pre-surgery		Surgery	Post-surgery						
**Assessment**	Screening	Enrollment		< = 48h post-op	2 weeks	6 weeks	3 months	6 months	9 months	12 months
Radiographs	●			●			●	●	●	●
Informed consent		●								
Serum creatinine		●		●	●					
Micro-costing			●	●						
Randomization			●							
Intervention			●							
Baseline data^a^				●						
Contact information				●						
EQ-5D				●			●	●	●	●
Planned endpoint assessment^b^					●	●	●	●	●	●
FIX-IT							●	●	●	●
WPAI						●	●	●	●	●
CRP					●	●	●	●	●	●
Adverse event screen					●	●	●	●	●	●

^a^Demographic, medical history, injury characteristics

^b^Fracture-related infection, unplanned fracture-related reoperation

Participants return to clinic for follow-up at 2 weeks, 6 weeks, 3 months, 6 months, 9 months, and 1 year following surgery (Table 5). Follow-up data collection is expected to conclude by January 2022. At the 2-week follow-up visit, the surgical wound is checked, and patients are assessed for surgical and medical complications. At all subsequent follow-up visits, clinical evaluation is performed, AP and lateral radiographs are taken, FIX-IT score is assessed, and EQ-5D and WPAI are administered.

Follow-up visits are conducted at a dedicated research clinic staffed by at least one study investigator and one research coordinator. In order to encourage follow-up attendance, patients are contacted by telephone twice in the week prior to scheduled appointments.

### Imaging analysis

Radiographs are uploaded to the secure online database where they are assessed for radiographic signs of healing using the mRUST score by three fellowship-trained orthopaedic trauma surgeons.

### Participant retention

In order to encourage adherence to follow-up, the following strategies are implemented:

1. All follow-up care is provided at no cost to study participants.
2. Follow-up visits take place in a dedicated study clinic to minimize wait times.
3. Three separate contacts are obtained for each patient upon enrollment. Contacts are notified of the patient’s participation in the study and encouraged to help the patient attend all follow-up visits.
4. Follow-up visit reminders are given via phone call and by text message during the week prior to the visit.
5. If patients are unable to attend an in-person visit at a designated study time point, data will be collected over the telephone.

These strategies were successfully utilized in our previous open tibia fracture trial performed through the same collaboration [12].

## Data management

Study data are collected and managed using REDCap electronic data capture tools hosted at the coordinating center [29, 30]. REDCap is a secure, web-based software platform designed to support data capture for research studies.

Patient privacy is protected using established data security practices including dual-authentication protection of data capture tools and data storage servers, de-identification of data prior to export, and data coding with destruction of the data key at the end of the study.

## Safety monitoring

An independent data safety and monitoring committee (DSMC) has been convened to monitor recruitment, retention, data quality, and patient safety. The DSMC is comprised of an orthopedic surgeon, an emergency medicine physician, and a trial methodologist who serves as chair of the committee. The committee consists of both American and Tanzanian members who have collective experience in the management of orthopaedic injury and conduct and monitoring of randomized clinical trials including in sub-Saharan Africa. The DSMC convenes at least every 6 months, with additional ad hoc meetings as needed.

Based on review of adverse events, the DSMC will terminate the trial prematurely if it determines the intervention is associated with harm at any time point, and findings will be reported to required parties and enrolled patients. Interim analyses of efficacy will not be conducted and stopping for treatment effect will not be performed for the pilot study.

## Statistical plan

### Sample size determination

The study described herein is a pilot trial with a target of 100 patients [46]. Current available data on rates of fracture-related infection are sparse and generated from dissimilar trials; therefore, the data from this pilot trial will be used to more accurately estimate sample size requirements for the subsequent definitive trial. The sample size (50 per group) provides approximately 80% power to detect a difference of 20% between the two arms (assuming 5% occurrence in the intervention group). If available at the time of study completion, data from pGO-Tibia will be pooled with other similar clinical trials using meta-analysis techniques.

### Statistical methods

Statistical methodology for the analysis of each feasibility outcome and planned primary and secondary endpoint is outlined in Table 4.

### Feasibility outcomes

The feasibility aims are (1) recruitment, as measured by number of patients enrolled per month, (2) retention, as measured by percent of patients that complete 1-year follow-up, and (3) data completeness of safety outcomes (including serum creatinine and adverse event screening), treatment group allocation, and FRI criteria. These outcomes are summarized using means and proportions, without the use of inferential statistics. Progression to the planned definitive trial is based on the satisfactory completion of these outcomes.

### Planned primary and secondary endpoint analysis

The planned primary endpoint is occurrence of fracture-related infection, a binary outcome. The planned primary analysis will be conducted as a binomial regression with the complementary log-log link, the estimated effect will be the relative hazard, and the analysis will be two-sided, with a type I error rate (alpha) of 0.05. The planned secondary endpoints are occurrence of nonunion, occurrence of fracture-related reoperation, health-related quality of life, radiographic healing, clinical healing, occurrence of suggestive FRI, lost productivity, and direct medical costs. Analyses will be conducted on an intent-to-treat basis. The planned secondary analysis of binary outcomes will be conducted as Fisher’s exact test on the 2 × 2 cross-tabulation out each outcome with treatment group assignment. Risk difference between treatment groups will be reported with 95% confidence interval as assessed by binomial regression with identity link. For continuous variables, means will be compared between treatment groups using two-tailed Student’s *t* test (alpha = 0.05).

The pilot trial as described is underpowered for hypothesis testing of planned primary and secondary endpoints. As such, the testing described herein are the planned analyses for the definitive trial and will not be used exclusively for sample size calculations, nor considered in progression criteria for the definitive trial.

## Dissemination

The results of this pilot trial will be submitted for publication. Anticipated publishable findings are the feasibility outcomes and planned primary and secondary endpoints including the rate of fracture-related infections, fracture healing, and health-related quality of life.

## Discussion

We describe the implementation of a pilot study to assess the feasibility of a single-center masked randomized controlled trial to compare the efficacy of locally applied gentamicin to placebo for the prevention of fracture-related infection in open tibial shaft fractures. This represents the first clinical trial evaluating locally applied gentamicin for open tibia fractures, and the first clinical trial with this degree of methodologic rigor for any intervention for open tibial fractures in a low-income country.

The strengths of this study include masking of participants and investigators, randomized allocation of treatment groups, and rigorous trial design. The masking protocol prevents bias in allocation and assessment, and randomization prevents confounding bias [47]. Further, the study addresses a locally relevant clinical problem with a potentially sustainable intervention. Prevalence of open tibia fracture is high in LMICs, and rate of deep surgical site infection has been estimated to be 20% in these cases. Locally applied gentamicin is a low-cost intervention that may lower the burden of fracture-related infection. Finally, the volume of open tibia fractures makes MOI a unique clinical environment with a high number of eligible patients and proven capacity to enroll, randomize, and follow-up patients with open tibial fractures.

A critical consideration for trials conducted in LMIC is the ethical concerns related to potential exploitation of economically disadvantaged populations. When done ethically, clinical research conducted in LMICs can benefit all stakeholders by providing a high volume of the condition of interest and by advancing locally relevant medical knowledge and treatments. Further, research partnerships between HICs and LMICs can build research and health care capacity in LMICs [48]. Exploitation may occur if the local population is unlikely to be offered or able to afford the intervention or treatment under study. This study is of value to public health in Tanzania because the intervention is an affordable and widely available single use medication that, if proven effective, could reasonably be adopted in Tanzania and other LMICs. This pilot trial was designed to satisfy ethical standards for clinical trials in LMICs by addressing a locally relevant disease with an affordable intervention [48]. Both US and Tanzanian collaborators stand to benefit from the results of this trial and its conduct in Tanzania. The volume of open tibia fractures is much higher in Tanzania than in the USA and Europe (Table 1), increasing the study feasibility. Tanzanians, specifically, may benefit from development of this affordable intervention in their setting where the burden of open tibia fracture is high, and such results may be generalizable to patients around the world.

Success of this protocol relies heavily on the established working relationship with all members of the local research team including specialist and resident surgeons, physicians, nurses, surgical staff, hospital administrators, and research coordinators. The integration of local stakeholders adds to the merit of this study in that it contributes to the building of lasting research capacity at MOI, further satisfying the recommendations for ethical conduct of research in LMIC [49].

## Conclusions

This study will provide important preliminary data to inform a larger definitive trial to study the use of intraoperative, locally injected gentamicin to prevent fracture-related infections following open tibia fractures. Ultimately, the success of the definitive trial could establish the evidence base necessary for the implementation of a sustainable, low-cost intervention to reduce the burden of infection after open tibia fracture in LMICs.

## Supplementary Information

**Additional file 1.** pGO Tibia Injection Procedure.mp4.

## Data Availability

Not applicable.

## Abbreviations

CRP: C-Reactive protein
DSMC: Data safety and monitoring committee
EQ-5D: EuroQol-5 Dimensions, 3-level questionnaire
FIX-IT: Function Index for Trauma
FRI: Fracture-related infection
HIC: High-income countries
LMICs: Low- and middle-income countries
MOI: Muhimbili Orthopaedic Institute
mRUST: Modified Radiographic Union Score for Tibia
MUHAS: Muhimbili University of Health and Allied Sciences
pGO-Tibia: A Pilot Masked, Randomized Controlled Trial Evaluating Locally-applied Gentamicin versus Saline in Open Tibia Fractures
REDCap: Research Electronic Data Capture
UCSF: University of California San Francisco
